# Exploring alterations in the gut resistome in medically treated inflammatory bowel disease patients

**DOI:** 10.1186/s12866-026-05101-9

**Published:** 2026-04-28

**Authors:** Jonas Christoffer Lindstrøm, Hilde S. Vollan Gjerdrum, Ola B. Brynildsrud, Tone Møller Tannæs, Anja Bråthen Kristoffersen, Petr Ricanek, Truls M. Leegaard, Jørgen Vildershøj Bjørnholt, Silje Bakken Jørgensen, Hege S. Tunsjø, Christine Olbjørn, Trond Espen Detlie, Jørgen Jahnsen, Vendel A. Kristensen, Marte Lie Høivik, Johannes R. Hov, Aina EFossum Moen, IBSEN-III Study group, AK Lunder, A Sagosen, F Vikskjold, T Tønnesen, A Hasund, J Pallenschat, K Schmidt, O Høie, S Andersen, T Bergene Aabrekk, MB Bengtson, V Strande, EE Løvlund, KA Holten, M Henriksen, G Huppertz-Hauss, H Yassin, R Boyar Cetinkaya, SO Frigstad, C Sommer, G Perminow, K Skram, I Johansen, AM Aas, S Hyll Hansen, C Lund, I Frivold Glad, I Zerouga, R Opheim, Ø Asak, CM Ystrøm, R Torp, HK Holm, Ø Hovde, M Maaseng, B Aballi, BC Olsen, J Valeur, LP Jelsness-Jørgensen

**Affiliations:** 1 Department of Method Development and Analytics, Norwegian Institute of Public Health, Oslo, Norway; 2 Division of Medicine, Department of Clinical Molecular Biology (EpiGen), Akershus University Hospital and University of Oslo, Lørenskog, Norway; 3 Department of Gastroenterology, Lovisenberg Diaconal Hospital, Oslo, Norway; 4 Department of Microbiology and Infection Control, Akershus University Hospital, Lørenskog, Norway and University of Oslo, Oslo, Norway; 5 Institute of Clinical Medicine, University of Oslo, Oslo, Norway; 6 Department of Microbiology, Oslo University Hospital, Oslo, Norway; 7 Department of Emergency Medicine, Akershus University Hospital, Lørenskog, Norway; 8 Department of Life Sciences and Health, OsloMet, Oslo, Norway; 9 Department of Pediatric and Adolescent Medicine, Akershus University Hospital, Lørenskog, Norway; 10 Department of Gastroenterology, Akershus University Hospital, Lørenskog, Norway; 11 Department of Gastroenterology, Oslo University Hospital, Oslo, Norway; 12 Norwegian PSC Research Center and Section of Gastroenterology, and Research Institute of Internal Medicine, Oslo University Hospital, Oslo, Norway

**Keywords:** IBD, AMR, Microbiome, Resistome

## Abstract

**Introduction:**

The members of the human gut microbiota contain a large diversity of genes, including antimicrobial resistance genes (ARGs) known as the gut resistome. The resistome is susceptible to alterations when compositional changes occur in the fecal and gut microbiome. Medical treatment may affect members of the gut microbiota. This study hypothesizes that medication used by patients with inflammatory bowel disease (IBD) leads to an increased prevalence and diversity of ARGs in the gut and a corresponding change in the taxonomic composition of the fecal microbiome.

**Methods:**

Fecal samples from 16 Crohn’s Disease (CD) and 16 Ulcerative Colitis (UC) patients, and 13 symptomatic controls (patients experiencing gastrointestinal symptoms, but with no endoscopic or histologic signs of IBD at inclusion, and no evidence of IBD during follow-up, were classified as symptomatic non-IBD controls) were subjected to metagenomic sequencing. The samples were collected before initiation of IBD medication, and after one year of treatment. Patients were treated with 5- Amino Salicylic Acid, Biological treatment, and Corticosteroids, or a combination of the three. Resistance Gene Identifier Comprehensive Antibiotic Resistance Database (RGI CARD) and regression modelling were used to analyze the abundance and diversity changes in the ARGs and the taxonomy.

**Results:**

We found significant associations with medicine use and abundance changes for eight resistance genes (Antibiotic Resistance Ontology (ARO) terms), four AMR gene families and 14 AMR drug classes. The use of 5-ASA was associated with abundance changes for the efflux pump efpA. This medication was also associated with significant changes in the “pyrazinamide resistant rpsA” gene family and with six drug classes (cephamycin, diaminopyrimidine, mupirocin, penem, pyrazinamide and rifamycin). Biological treatment was associated with changes in abundance of five drug classes (Zoliflodacin, lincosamide, macrolide, streptogramin and tetracycline). Corticosteroids were associated with changes in the ARO terms sul2, OXA beta-lactamase AMR gene family, and three drug classes (carbapenem, glycylcycline, and triclosan).

**Conclusions:**

All IBD medication groups were found to be associated with significant abundance changes within the fecal resistome between inclusion and follow-up time points, where corticosteroid treatment resulted in less resistance in the microbiota compared to in the persons not treated with corticosteroids (either 5-Aminosalicylic Acid or Biological treatments).

**Supplementary Information:**

The online version contains supplementary material available at 10.1186/s12866-026-05101-9.

## Introduction

The gut microbiome is a complex ecosystem in the intestinal tract consisting of an estimated 10^13^ microbes and studies have suggested that as many as 20% of these possess antimicrobial resistance genes (ARGs) [1, 2]. In the human gut a large diversity of ARGs exists both among the commensal and the opportunistic members of the microbiota [3]. The resistome holds a collection of ARGs, together with antiseptic resistance and other resistance genes [4]. Even though ARGs can be exchanged between bacterial phylogenetical lineages, the distribution of ARGs is largely constrained by phylogeny, and changes to the microbiome will thus affect the resistome [5, 6]. Findings demonstrate that the diversity of the microbiome and the associated resistome is reduced immediately after being exposed to antibiotics [7]. A return to a pre-antibiotic microbial diversity state is generally observed some months after the end of antibiotic exposure, but an altered taxonomic state and an increase in ARGs have been demonstrated [8].

Non-antimicrobial medication may also affect the gastrointestinal microbiota by pushing it to a more resistant state [9]. Studies have shown that bacteria possessing antimicrobial resistance (AMR) mechanisms and genes are less affected by non-antimicrobial therapy, whereas non-resistant strains can be growth restricted or even killed [10]. AMR genes not only provide resistance to antibiotics but may also increase bacterial robustness against other therapeutic interventions by modifying membrane permeability, efflux pump activity, and stress response pathways [11].

Inflammatory bowel disease (IBD) is a chronic relapsing disease affecting different areas of the gastrointestinal tract. It consists of mainly two clinical entities, Crohn’s disease (CD) and ulcerative colitis (UC). The disease pathophysiology is not entirely elucidated, but genetics, environmental factors, aberrant immune reaction against gastrointestinal microbiota and the microbiota itself are all assumed to play a role [12]. Patients suffering from IBD have an increased risk of serious and opportunistic infections [13–15]. The increased risk is related to the disease itself as well as to the medical therapy given. IBD patients are exposed to drugs which may affect the microbiome and the gut resistome [16, 17]. However, studies on the IBD resistome and the effect of IBD medications are limited [18].

Gut microbiome studies of CD and UC have revealed a state of dysbiosis [19]. It is characterized by a decrease in microbial diversity and an increase in pathogenic bacteria including members of the *Enterobacteriaceae* family [20]. *Enterobacteriaceae* like *E. coli, Klebsiella, Salmonella* and *Shigella* are pathogens known to rapidly acquire ARGs [21]. A dysbiotic gut microbiome with a high incidence of ARGs is expected to have consequences for the antimicrobial treatment of infections resulting in a need for more broad-spectrum antimicrobial therapy [22–24]. Increased prevalence of AMR will also lead to increased costs due to medication that is more expensive, longer hospitalizations and implementation of infection control measures [25]. Furthermore, as IBD is a chronic disease, these patients often require continuous medical therapy, with the need for treatment optimization and change of therapeutic agents. How this constant intake and change of medications affect the gut resistome is not well described, but a drive towards an increase in the prevalence and diversity of ARGs is plausible. Growing evidence indicates that several commonly used drugs, including NSAIDs, lipid-lowering drugs, and β-blockers, can alter gut microbiota and induce selective pressures that promote ARG enrichment and horizontal gene transfer within the gut microbiome [26–29]. These non-antimicrobial treatments may contribute to shaping gut resistome in IBD patients, independent of antibiotic exposure. For example, NSAIDs can select resistance mechanisms that also confer antibiotic resistance, such as multidrug efflux pumps [29]. Once established, these mechanisms can create an environment conducive to the development and dissemination of AMR [29]. The present study investigates the effect of non-antimicrobial IBD medication on the diversity and abundance of ARGs in the fecal microbiome of IBD patients through metagenomic analysis and bioinformatic modeling. Our hypothesis posits that IBD medication contributes to an altered diversity and presence of ARGs, reflecting changes in the fecal microbiome. These processes are particularly relevant in IBD patients who often undergo continuous and varied medical treatment.

## Material and methods

### Patient inclusion, sample collection and follow-up

Patients included in the present study are part of the IBSEN III study [30], a prospective ongoing study of patients with suspected IBD referred to the hospitals in the South-Eastern health region of Norway between 2017 and 2019. The diagnosis of IBD was based on internationally accepted diagnostic criteria [31, 32]. Patients with gastrointestinal symptoms, but with no endoscopic or histologic signs of IBD at inclusion, and no evidence of IBD during follow-up, were classified as symptomatic non-IBD controls, herby referred to as controls. With regards to the use of medications in the control group, they did not receive any IBD medication, but we do not have any data on whether they used other over-the-counter medicine or used medication prescribed from their general practitioner. The patients provided stool samples at inclusion and at the one-year follow-up. IBD treatments were only commenced if diagnosis were confirmed at inclusion. In total, 45 patients with two fecal samples (at start and follow-up) were included in our analysis.

In the present study, IBD patients and controls were selected based on the following criteria: age ≥ 18 years old, provided stool samples at both inclusion and follow-up, and no use of antimicrobial therapy the last 12 weeks prior to inclusion or during the follow-up period.

Stool samples were collected by the patients at home using the Protocult stool sampling device (Ability Building Center, Rochester, MN, USA) in tubes with a preservative liquid (Stool Collection Tubes with Stool DNA Stabilizer (Stratec Molecular GmbH, Berlin, Germany)) [33], according to a protocol consistent with the requirements of the tube manufacturer, which describes that the DNA stabilizer allows room temperature storage up to 3 months. Still, for optimal quality, the participants were instructed to keep the samples in the refrigerator until mailing to a central lab for biobanking, ideally within a day of collection. Long-term storage was at − 80 °C until DNA purification.

### Clinical parameters and medical therapy

All clinical parameters and given medical therapy were collected in the IBSEN III study as described previously [30]. IBD medication was classified in three groups: 1) 5-aminosalicylic acid (5-ASA); 2) Biological treatment consisting of combination therapy with anti-TNF (infliximab, adalimumab, golilumab) and immunomodulators (azathioprine, methotrexate); 3) Corticosteroids (prednisone, budesonide). IBD patients received medical therapy decided by the treating physician. The IBD patients in the present study might have received either several consecutive treatments or combinations of treatment between inclusion and follow-up. Due to the small sample size this has not been adjusted for in our analysis.

### DNA purification, library preparation and metagenomic sequencing

Microbial DNA was extracted using the PSP Spin Stool Plus DNA Kit (Stratec Molecular GmbH) with a protocol modified by adding a bead-beating step, as described elsewhere [34]. The concentration and the quality of all DNA samples were measured using the NanoDrop-1000 and agarose gel electrophoresis.

Metagenomic libraries were generated at the Norwegian Sequencing Centre (NSC, Oslo, Norway) using Swift 2S® Sonic DNA Library Kit (Swift Biosciences, Integrated DNA Technologies Inc, Iowa, US). Metagenomic sequencing was performed at NSC on Illumina NovaSeq S4 set at 150 bp paired-end reads according to the standard procedures at the NSC.

### Data processing and analyses

Human reads were removed using the desensitize workflow FHI desensitize (https://gitlab.com/uit-sfb/fhi-desensitize), created for the Norwegian Institute of Public Health [35]. The full MetaWRAP v1.3 pipeline was used for read quality control, metagenomic assembly, taxonomic analysis, binning and functional annotation [35]. Kraken2 was used for taxonomic classification, using the “Standard” build (containing data from RefSeq Bacteria, Archaea, Viral, Plasmid, Human and UniVec_Core) as of August 2022. MetaWrap default filtering process and pipeline parameters can be found here: https://github.com/bxlab/metaWRAP/blob/master/Module_descriptions.md. The counts of mapped genera per sample from Kraken2 (embedded in the MetaWRAP pipeline) can be found in Supplementary Table 4.

The ARGs present in the microbial reads (contigs) were predicted using the Resistance Gene Identifier (RGI) *bwt* pipeline (https://card.mcmaster.ca/analyze/rgi). The RGI *bwt* was run using trimmed metagenomic reads from MetaWRAP as input, default settings and Bowtie 2 to align the reads to the Comprehensive Antibiotic Resistance Database (CARD) v/3.1.4 and to the CARD’s resistomes and variants dataset v/3.0.9 [36]. The predicted ARGs are placed in a hierarchical structure of gene ontology terms where the present study uses the primary ontology, the Antibiotic Resistance Ontology (ARO), AMR gene families, drug classes and resistance mechanisms. The resulting counts of mapped genes per sample can be found in Supplementary tables 2–3. An average mapping quality (MAPQ) score > 10, percent coverage ≥ 50% and average depth ≥ 10 were applied.

### Statistical methods

To analyze the association between medicine use and changes in the resistome and microbiome, we used log-linear negative binomial regression models. Separate models are fitted for each outcome (CARD ARO terms, AMR Gene Gamily, Resistance mechanism, Drug class or genus) and medication. Let ${\upmu }_{ijk}$ be the log-expected counts at follow-up for individual i, for the outcome j in the model for medication k. Then the model is

$$\begin{aligned} {\upmu }_\mathrm{ijk}=&{\upbeta }_\mathrm{0jk}+log\left(\mathrm{T}_\mathrm{i}\right)+{\upbeta }_\mathrm{1j}\mathrm{x}_\mathrm{ij}\\&+{\upbeta }_\mathrm{jD}\mathrm{D}_\mathrm{i}+{\upbeta }_\mathrm{jk}\mathrm{M}_\mathrm{ik}\\&{\mathrm{y}}_{\mathrm{i}}\sim \mathrm{NegBin}\left(\mathrm{e}^{{\upmu }_\mathrm{ijk}},{\upsigma }_\mathrm{jk}\right) \end{aligned}$$

where ${\upbeta }_{0jk}$ is the intercept, $log\left({T}_{i}\right)$ is the logarithm of the total number of reads in the follow-up sample of individual i, xi is the relative abundance of the outcome at baseline (the number of counts divided by total reads), D_i_ is the diagnosis indicator (CD, UC, and symptomatic control), and M is the indicator for medication use for the k’th medication. y_i_ follows a Negative Binomial distribution with expected value $exp\left({\upmu }_{ijk}\right)$ and dispersion parameter ${\upsigma }_{jk}$. In case the model fitting procedure did not converge, a Poisson model was used instead, fitted using a quasipoisson likelihood. ${\upbeta }_{jk}$ is the parameter of interest as it relates the medication use with the outcome. In the analyses of 5-ASA, the CD and UC diagnoses were merged because all UC patients used 5-ASA.

Any significant association between medication and changes in the ARG abundance or taxa abundance between inclusion and follow-up are given as either positive or negative. A positive association means that the *change* (in abundance of ARG or taxa) from inclusion to follow-up is greater among patients using medicine vs those that did not receive this medication. A negative association means that the *change* from inclusion to follow-up is less among medication users than among non-users.

To adjust for multiple hypothesis testing we used False Discovery Rates (FDRs) that were calculated separately for each feature class (ARO terms, AMR gene family and Drug class etc.). FDR < = 0.1 was considered statistically significant, meaning that 10% of the significant hits within the feature class can be expected to be false positives. In analyses where only one response was tested, we report the unadjusted *p*-values. To ensure robustness of significant findings, the medication use was permutated, and the analysis was repeated 100 times. Findings where the *p*-value from the permuted analysis was less than the real *p*-value for at least 10 of the 100 runs were thought to be significant due to outliers and disregarded. Resistance genes (ARO terms), AMR gene families, drug classes and resistance mechanisms with at least 100 reads in total were used in regression analyses for associations with medicine use. Associations between taxonomic profile and medication used were analyzed at the genus taxonomic level. We performed regression analysis only on those genera that had an abundance greater than 0.5% in at least five samples. This was done to avoid issues with non-convergence during the model fitting process.

To make comparisons of the overall composition of ARO-terms in a multivariate fashion we used the Bray–Curtis dissimilarity index together with multidimensional scaling and distance-based analysis of variance. Counts were divided by the total number of reads in the sample before the analysis. We also compared the within-patient dissimilarities between the two timepoints and used a Wilcoxon test to test for associations with medicine use. Bray–Curtis dissimilarities on the relative abundances were also used for beta-diversity analysis of taxa composition. The Shannon index was used to measure alpha diversity, using data on the genus taxonomic level. We tested differences at inclusion using linear regression, and changes within diagnosis groups using paired t-tests. Association with medicine use were analyzed using linear regression models similar to the ones used to analyze ARG and taxa. An analysis of the association between alpha diversity and total ARG content in each was done using a negative binomial regression analysis with ARG abundance a s response, with log total read counts as offset and the Shannon index as the only predictor.

Statistical analyses were performed in the R statistical framework (version 4.1) [37]. The vegan package was used for the beta-diversity analyses.

## Results

In total, 110 samples from 59 patients were subjected to DNA extraction and sequencing, and all samples passed extraction and sequencing quality control. Fourteen patients, 20 samples, were excluded after sequencing due to the use of antibiotics after inclusion, or because the patients were represented with only one sample, giving 45 patients included in the analyses. Sixteen patients were diagnosed with CD, 16 with UC and 13 were in the control group (Table 1, Supplemental Table 1). All CD and UC patients received IBD medication between inclusion and follow-up (Supplemental Fig. 1). None of the controls received IBD medication.

**Table 1:** Demographics showing diagnosis and medical treatment between inclusion and follow-up

	**CD**	**UC**	**Controls**	**Total**
Patients (n)	16	16	13	*45*
IBD medication				
5-ASA	3	16	0	19
Biological treatment	9	2	0	11
Corticosteroids	10	6	0	16

The average total number of reads per sample was 31,266,616 (ranging from 86,174 to 74,446 882), where on average 0.7% of the reads were mapped to an AMR related sequence in CARD (ranging from 0.5–2.6%). In total, 1105 CARD ARO genes, 174 Gene Families and 42 Drug classes were identified, and after excluding those that were represented by fewer than 100 reads in total, 505 ARO genes, 117 gene families, 12 resistance mechanisms, and 39 drug classes were further analyzed. The average depth was 9,431,222,940 (min = 25,852,200).

### IBD medication associated in changes in the gut resistome

**Fig. 1:**
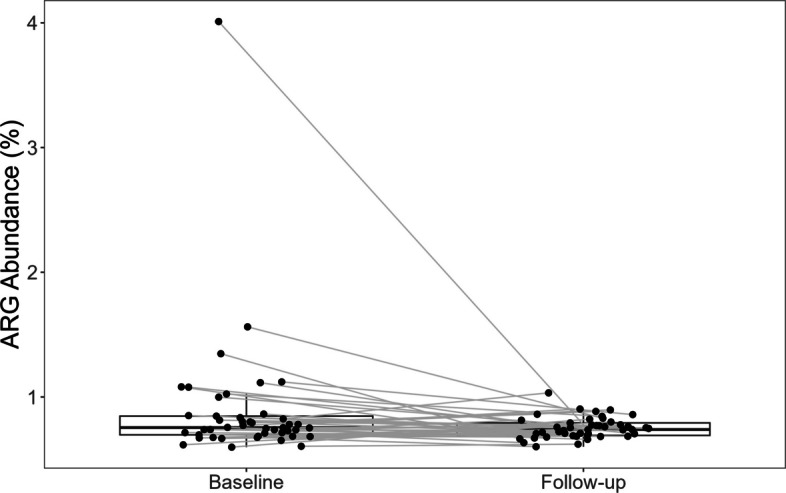
Boxplot of total ARG abundance at baseline and follow-up. Total ARG abundance (y-axis) is the percentage of reads that were mapped to an ARO term from the CARD RGI database. The boxplot is overlaid by points showing the individual samples, at two time-points (x-axis). No significant change was seen between the two time points (*p* = 0.12, paired Wilcoxon test)

The average abundance of all predicted ARGs decreased non-significantly from baseline (0.89% of reads) to follow-up (0.75%, p = 0.12, paired Wilcoxon test, Fig. 1). IBD medication was associated with a non-significant decrease in total AMR gene abundance (Fold changes in the range 0.92–0.97, Supplemental Table 5).

Table 2 lists the significant associations (FDR ≤ 0.1) between medicine use and changes in abundance in three classes of CARD RGI annotations. We found that changes in eight resistance genes, four AMR gene families and 14 AMR drug classes were significantly associated with medicine use (See Supplemental Tables 6–9 for results for all analyses). Some of the genes were annotated to confer resistance due to point mutations, but because of sequencing depth these results must be interpreted with caution. Sequencing depth was normalized to a minimum of 10 reads per gene to ensure robust gene-level detection and reduce false positives associated with platform-specific substitution error [38]. For resistance mechanism annotations, no significant associations were found.

**Table 2:** Association between medical therapy and changes in resistance genes from inclusion to follow-up. The table shows the estimated associations between medicine use and changes in the counts of reads annotated with three different annotation classes from the CARD RGI database. Only statistically significant (FDR ≤ 0.1) associations are shown. The complete results of analyses are found in Supplementary Tables 6–9

CARD RGI annotation class	Medication	Annotation	Relative difference in abundance change (95% CI)*	FDR
Resistance Gene (ARO term)	5-ASA	Mycobacterium tuberculosis rpsA mutations conferring resistance to Pyrazinamide	3.40 [1.64—6.90]	0.091
	5-ASA	Mycoplasma Genitalium GyrA mutation confers resistance to fluoroquinolones	14.48 [3.44—58.94]	0.074
	5-ASA	efpA	5.03 [1.96—12.56]	0.091
	Biologicals	CrcB	0.17 [0.08—0.33]	0.003
	Biologicals	Mycobacterium intracellulare 23S rRNA with mutation conferring resistance to azithromycin	0.71 [0.58—0.87]	0.091
	Biologicals	Mycobacteroides abscessus 16S rRNA mutation conferring resistance to tobramycin	0.75 [0.64—0.87]	0.067
	Biologicals	Mycobacteroides chelonae 16S rRNA mutation conferring resistance to amikacin	0.77 [0.66—0.90]	0.091
	Corticosteroids	sul2	0.06 [0.01—0.48]	0.076
AMR Gene Family	5-ASA	16S rRNA methyltransferase (G1405)	0.35 [0.27—0.47]	0.000
	5-ASA	pyrazinamide resistant rpsA	3.40 [1.64—6.90]	0.078
	Biologicals	16S rRNA with mutation conferring resistance to pactamycin	8.29 [1.74—40.30]	0.079
	Corticosteroids	OXA beta-lactamase	0.19 [0.07—0.47]	0.062
Drug Class	5-ASA	cephamycin	0.31 [0.16—0.56]	0.019
	5-ASA	diaminopyrimidine_antibiotic	1.81 [1.26—2.57]	0.028
	5-ASA	mupirocin	2.97 [1.27—6.83]	0.085
	5-ASA	penem	0.51 [0.30—0.85]	0.085
	5-ASA	pyrazinamide	3.40 [1.64—6.90]	0.028
	5-ASA	rifamycin_antibiotic	1.27 [1.07—1.49]	0.066
	Biologicals	Zoliflodacin	1.61 [1.11—2.36]	0.085
	Biologicals	lincosamide_antibiotic	0.82 [0.72—0.95]	0.085
	Biologicals	macrolide_antibiotic	0.86 [0.76—0.96]	0.085
	Biologicals	streptogramin_antibiotic	0.84 [0.74—0.95]	0.066
	Biologicals	tetracycline_antibiotic	1.18 [1.05—1.34]	0.085
	Corticosteroids	carbapenem	0.43 [0.24—0.77]	0.066
	Corticosteroids	glycylcycline	0.30 [0.16—0.59]	0.028
	Corticosteroids	triclosan	0.59 [0.40—0.88]	0.086

^*^Negative Binomial regression

**Fig. 2:**
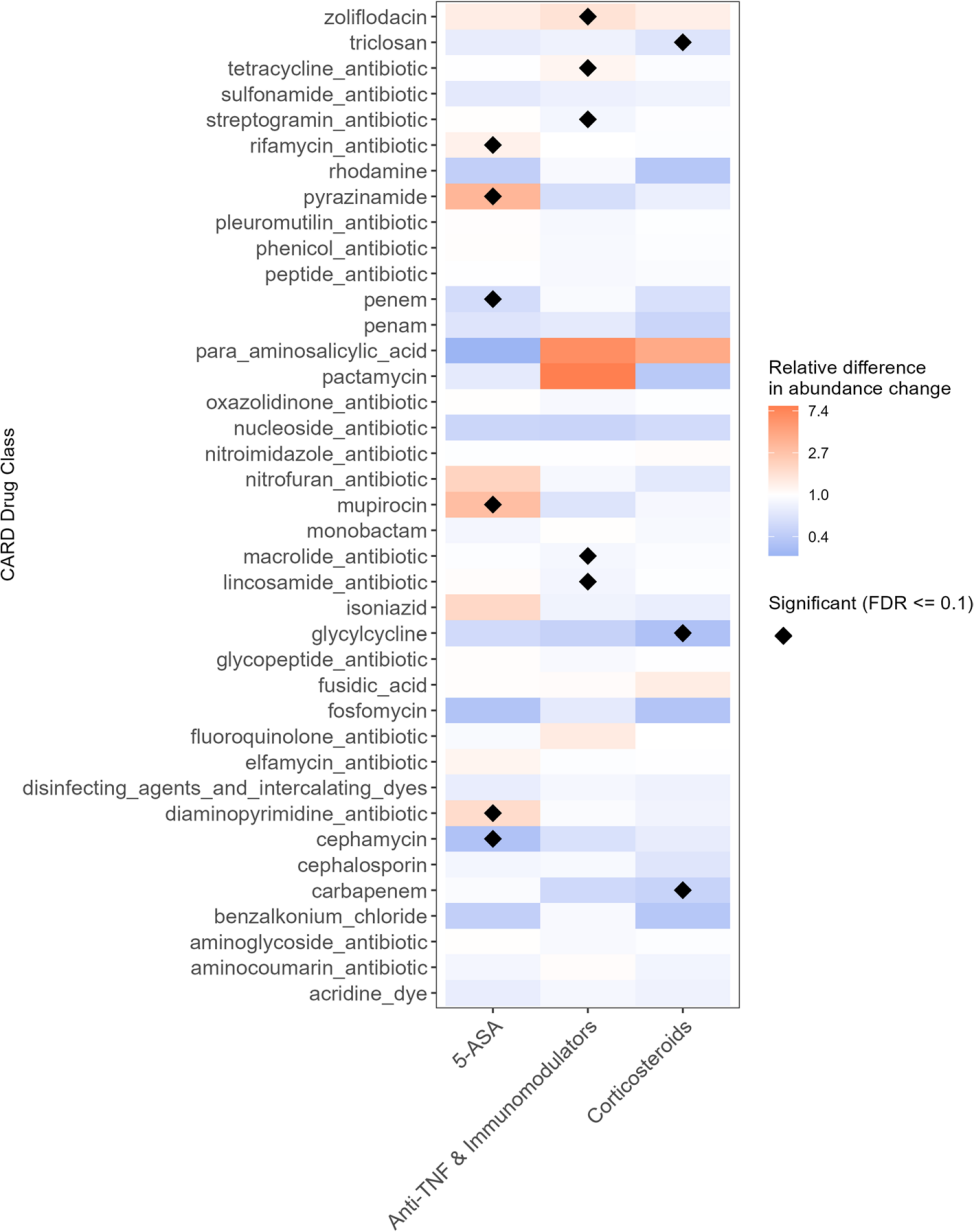
Medicine use and drug classes. Associations between medicine use (x axis) and changes in abundance of genes annotated with conferring resistance to drug classes (y axis). The strength and direction of each association is color coded with blue indicating a negative association, red a positive association, and white is used when there is no association. Statistically significant (FDR < 0.1) associations are highlighted with a black diamond

The use of 5-ASA was significantly (FDR ≤ 0.1) associated with abundance changes in three resistance genes, two AMR gene families and six drug classes (Table 2, Fig. 2, Supplemental Tables 6–9, Supplemental Fig. 2A −2Z). Biological treatment was significantly associated with abundance changes of four resistance genes, one AMR gene family and five drug classes (Table 2, Fig. 2, Supplemental Table 6–9, Supplemental Fig. 2A – 2Z), while corticosteroid use was associated with changes in one AMR gene family and three drug classes (Table 2, Fig. 2, Supplemental Table 6–9, Supplemental Fig. 2A - 2Z).

**Fig. 3:**
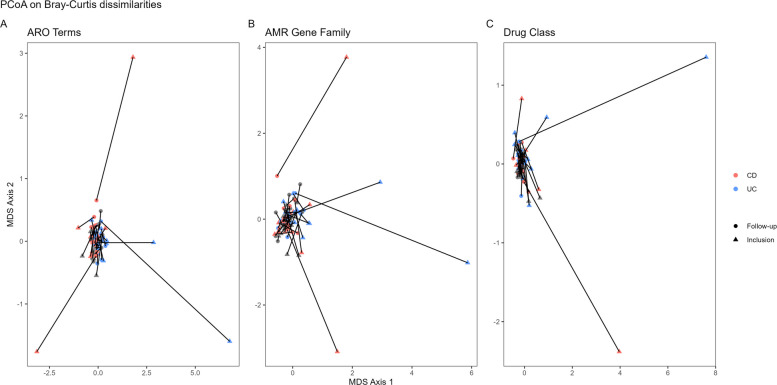
Bray–Curtis analysis. Pairwise Bray–Curtis dissimilarities were computed for each sample based on the abundance of CARD annotation categories and are here shown as multidimensional scaling plots. Each subplot shows the analysis for a different annotation category: **A** Resistance genes (ARO term) **B** AMR Gene Family **C** Drug Class. Paired samples from the same patient are connected with a line, and the diagnosis of the patient is color coded, and the timing of the sample is indicated by differently shaped points. Most of the samples form a single cluster, except for a few outliers, without any separation between diagnosis groups. All outlier samples were collected at inclusion, and the outliers in the three annotation categories are the same samples. The samples from these patients become similar to the rest of the samples at follow-up

The Bray–Curtis analyses of the predicted ARGs, AMR gene family and drug class shows little clustering in terms of diagnosis or sampling time (Fig. 3A - C). PERMANOVA R-squared showed that diagnosis and sampling time accounted for < 5% of the variation between samples, while the patient ID accounted for 55%—63% of the variation (Supplemental Table 11). When comparing the within-patient dissimilarities from inclusion to follow-up, there were no significant associations with medicine use (Supplemental Table 11).

### IBD medication associated with microbial composition

**Fig. 4:**
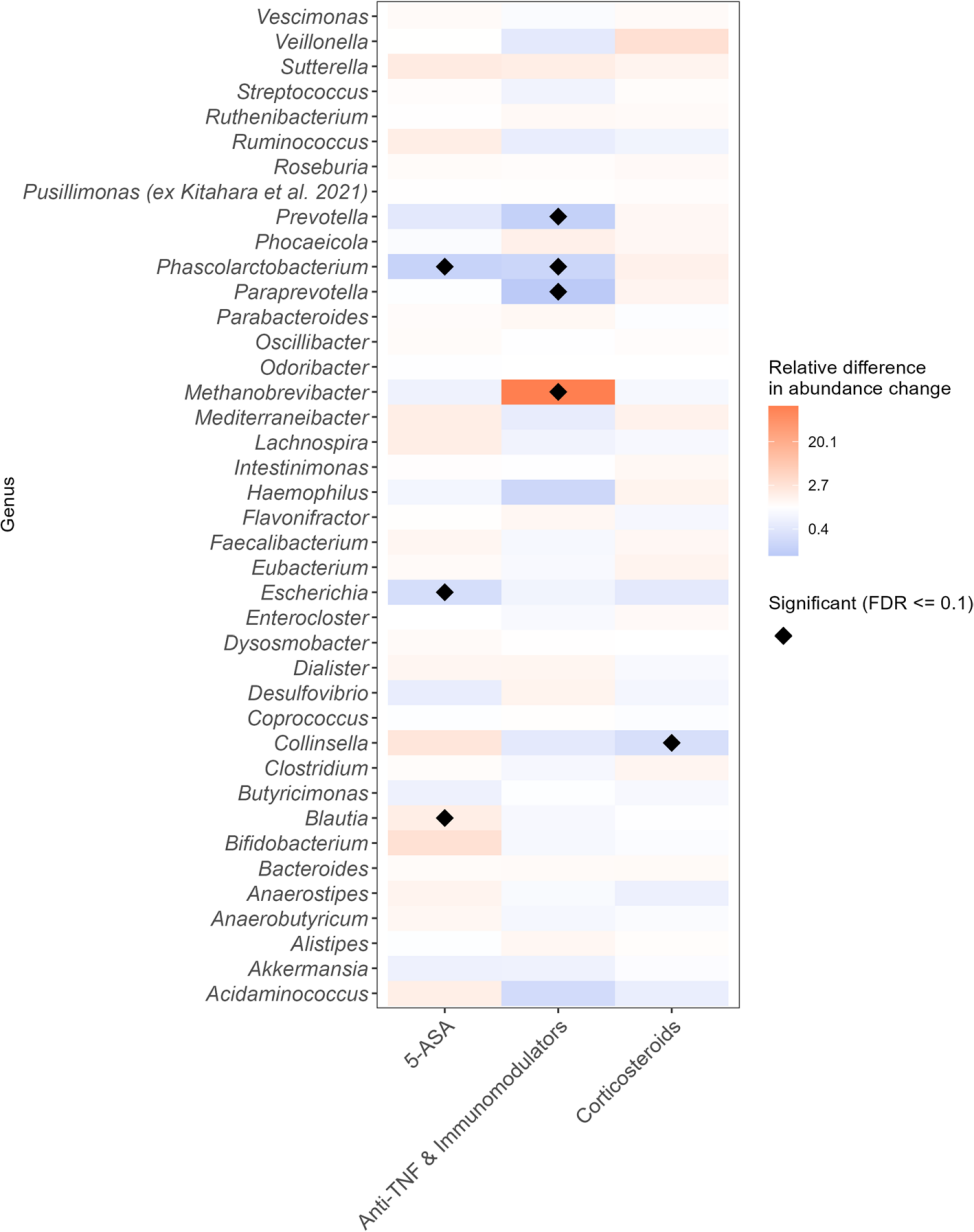
Medicine use and genus abundance. Associations between medicine use (x axis) and changes in genus abundance (y axis). The strength and direction of each association is color coded with blue indicating a negative association, red a positive association, and white is used when there is no association. Statistically significant (FDR < 0.1) associations are highlighted with a black diamond

To better characterize our metagenomic dataset and to complement the ARG analyses we also examined the association between medicine use and taxonomic composition. We found that medicine use was associated with changes in a few genera (Table 3, Fig. 4, Supplemental Table 10). 5-ASA use was positively associated with the genus *Blautia* when compared to patients not using 5-ASA (Table 3, Supplemental Fig. 3A). The use of biological treatments was positively associated with the genus *Methanobrevibacter* and negatively associated with the genera *Paraprevotella, Prevotella,* and *Phascolarctobacterium* (Table 3, Supplemental Fig. 3D - G). The use of corticosteroids was negatively associated to the genus *Collinsella* (Table 3, Supplemental Fig. 3H).

**Table 3:** Association between medical therapy and changes in genus abundance from inclusion to follow-up. The table shows the estimated associations between medicine use and changes in genus abundance. Only statistically significant (FDR ≤ 0.1) associations are shown. The complete results of analyses are found in Supplementary Table 9

**Medication**	**Genus**	Relative difference in abundance change (95% CI)*	**FDR**
5-ASA	*Phascolarctobacterium*	0.16 [0.06—0.40]	0.005
	*Escherichia*	0.25 [0.09—0.64]	0.056
	*Blautia*	1.87 [1.21—2.86]	0.056
Biologicals	*Methanobrevibacter*	101.96 [13.95—753.04]	0.000
	*Paraprevotella*	0.11 [0.04—0.33]	0.002
	*Prevotella*	0.14 [0.05—0.53]	0.013
	*Phascolarctobacterium*	0.18 [0.07—0.57]	0.035
Corticosteroids	*Collinsella*	0.26 [0.13—0.51]	0.005

^*^Negative Binomial regression

At inclusion, CD patients had significantly lower alpha diversity than controls (*p* = 0.037), but no significant difference were seen between UC and controls (*p* = 0.68) (Supplemental Fig. 4 A). There was a significant increase in alpha diversity among CD patients from inclusion to follow-up (*p* = 0.04), but no significant change for UC and controls (*p* > 0.3). No significant associations were found with medicine use and changes in alpha diversity (Supplemental Fig. 4 B). There was a significant negative association between alpha diversity and total AMR gene abundance (Foldchange = 0.85, *p* = 0.001, negative binomial regression).

The multidimensional scaling plot of the Bray–Curtis dissimilarities revealed no separation between samples from the three diagnosis groups nor between the samples from the two sample time points (Supplemental Fig. 5). PERMANOVA R-squared showed that the main important factor that could explain the dissimilarities between samples was the patient ID, which could explain 72%, while diagnosis and sampling time accounted for less than 6% of the dissimilarities.

## Discussion

This study is, to our knowledge, the first to investigate the effect of non-antimicrobial IBD medication on antimicrobial resistance in the gut microbiome of IBD patients. We found that all IBD medication groups were found to be associated with significant abundance changes within the fecal resistome.

The observed changes were predominantly within the CARD drug classes, suggesting an accumulative effect of multiple single ARGs. While individual ARGs did not show significant differences, their combined effect contributed to the overall pattern. The dynamics of ARGs involve both the accelerated dissemination of certain resistance genes and the loss of others, resulting in some genes becoming more prevalent while others diminish. The average abundance of all predicted ARGs in the present study showed a decreasing trend, but within medical treatment groups, some ARGs increased significantly. Thus, an overall decrease can hide the increase of specific resistance genes.. This complex interaction underscores the multifaceted nature of ARG dynamics and their impact on resistance profiles. The result of the present study suggests that the three categories of medical treatment exerted different pressures on the ARG abundances, with the abundance being primarily positively associated with the use of 5-ASA and negatively associated with the use of corticosteroids. Biological treatment revealed both positive and negative associations to the ARG abundances.

Drug-microbiota interactions have a bidirectional effect where different taxa in the microbiota can either enzymatically activate or deactivate IBD treatments. Furthermore, studies have found that IBD treatments are implicated in altering intestinal inflammation through changing both microbiota composition and function. Studies have identified changes in microbial diversity and/or relative abundances of various microbial taxa. The pharmacomicrobiomics is extensively reviewed by Becker et al. [12].

ARGs are part of the microbial defense system, and several reviews exist on how antibiotics shape the gut microbiome [21, 39, 40]. Some resistant genes can be present in a multitude of bacterial genera whereas others can be found in one or few specific bacterial species [4]. Becker et al. discuss that the influence of microbiota on drug metabolism, xenobiotics (non-antibiotic and antibiotic drugs) can also modify intestinal microbial composition and function by selectively inhibiting certain taxa [12]. Microbiological stress induced by e.g. medical therapy could result in the enhancement of gene exchange within and between microbial species and an accelerated dissemination of ARGs [12].

Significant changes in the resistance gene abundances were detected in single resistance genes, AMR gene families and drug classes for patients within all medical therapy groups from inclusion to follow-up. The changes were mainly towards a reduction in the abundance of resistance genes, even though particular medicines were associated both positively and negatively with ARG abundance. Of note, corticosteroid treatment was exclusively negatively associated with ARG abundance.

The use of 5-ASA, the corner stone drug in the treatment of mild to moderate UC, was associated with a number of abundance changes among resistance genes and drug classes, and 5-ASA use also revealed a number of positive associations. 5-ASA is the first-line therapy for UC patients. In the present study all UC patients were prescribed 5-ASA therapy, which means that it is difficult to separate the effect of the drug from and inherent effect of the disease. Nevertheless, positive associations between 5-ASA use and the ARGs indicate an increase in abundance of resistance genes, which is of concern and should be investigated further in a larger study.

Potentially, our results from the 5-ASA use could lead to antibiotic treatment challenges when IBD patients are introduced to immunosuppressive drugs and develop infections. The presence of ARGs might require the use of more broad-spectrum antibiotics, which in turn might lead to more antibiotic resistance in the individual or in the community. Resistance mechanisms coded by the genes having positive associations to 5-ASA use are diverse, ranging from efflux pumps conferring multi-resistance (*efpA*) and specific gene mutations resulting in resistance (*rpsA, gyrA*) [41–43]. These ARGs can be found integrated chromosomally associated to transposons and integrons, but are also found to be carried on plasmids, thereby prone to horizontal gene transfer [3, 44, 45]. The resistance genes mentioned are found within a wide range of bacterial taxa, ranging from clinically relevant pathogens to species not traditionally found to cause any disease in humans [3, 44, 45]. The latter could potentially act as a resistance pool from which pathogens can acquire ARGs when needed. Similarly, within the drug classes having positive associations to 5-ASA use, a wide variety of antimicrobial agents were found, such as pyrazinamide (antimycobacterial agent), mupirocin (protein synthesis inhibitor), diaminopyrimidine (enzyme inhibitor) and rifamycin (RNA polymerase inhibitor). Two drug classes, the β-lactam drug classes cephamycin and penem, were negatively associated with the use of 5-ASA, showing a decrease in relative abundance from inclusion to follow-up. With β-lactam drugs being clinically important and commonly prescribed for diverse bacterial infections, the latter findings are considered positive.

The use of non-antimicrobial IBD medication has been found to alter the taxonomic composition of the gut microbiota [12, 46]. Only *Blautia* was found to be positively associated with 5-ASA treatment, despite the relatively large number of associated ARGs. According to Romero-Rodrigez et al*.*, *Blautia* is associated with a healthy adult gut and prominent in the intestinal microbiota [47]. The use of 5-ASA revealed changes in microbiota diversity, a result that agrees with resistance genes not being evenly distributed among members of a genus or species. Resistant members could be favored at the expense of the sensitive members belonging to the same genus or species.

In the biological treatment group, more than 70% of the patients were on active treatment when the follow-up samples were collected. Both positive and negative associations between biological therapy and ARGs changes were identified. A shift was seen towards more ARGs against the drug classes tetracycline, the experimental drug Zoliflodacin, and less resistance against macrolide, lincosamide and streptogramin.

In the taxonomic analyses, negative associations to biological therapy were discovered for *Paraprevotella* and *Prevotella* [12]. These two genera are part of the normal gastrointestinal gut microbiota and species within these genera have been reported to be reduced in the gut microbiota of IBD patients [48–50]. Like some other species in the gut microbiota, *Prevotella* carry resistance genes, including a variety of erm genes involved in MLS resistance [44]. This could be part of the explanation with regard to the observation of less resistance towards the MLS drug classes. *Paraprevotella* and *Prevotella* have been found to be linked to, and carry resistance genes, against tetracyclines [51, 52]. This contrasts the result to the positive association of ARGs grouped into the tetracycline CARD drug class but can be explained by the many species within these genera not carrying tetracycline resistance genes and by the many more bacterial taxa among the gut commensals carrying resistance genes for tetracyclines [39]. A positive association and an increase in abundance was discovered for the archaeal methanogen *Methanobrevibacter*, a genus found to be highly resistant to antibiotics including tetracycline and aminoglycosides [40]. *Methanobrevibacter* could be an emerging pathogen, thus a positive association and increase in relative abundance following biological therapy should be investigated further [53]. Studies have implicated *Methanobrevibacter* as a severe pathogen in IBD and other infections, however, there have been contradicting findings [54–57]. It is important to note that the distribution of ARGs and taxa varies greatly within the gut microbiota, highlighting the need for more research in this field to better understand these dynamics and their implications [58].

Only negative associations were found between the use of corticosteroids and the resistance genes abundances and bacterial genera. These are positive findings with regards to a lesser resistant gut microbiota. In the taxonomic analyses, the use of corticosteroids was negatively associated with *Collinsella*, a genus described as part of the disturbed gut microbiota [59]. This is in line with the study of Carstens and colleagues [60], where an association between active disease, corticosteroid therapy and a reduced abundance of *Collinsella* was found. *Collinsella* has been found to carry multiple resistance genes, among them genes conferring resistance to β-lactam antibiotics, tetracycline and multidrug resistance efflux pumps [61]. The observed decrease in relative abundance of ARGs and the decrease of *Collinsella* could thus be the result of a reduction of pathogenic resistant microbes and a microbiota returning to a healthier state.

### Limitations

The present study has limitations. Firstly, the absence of healthy controls restricts the generalizability of the findings to the broader population. Secondly, the metagenomic sequencing depth may not have been sufficient to detect all relevant single nucleotide polymorphisms (SNPs), also known as mutations, impacting the reliability of SNP detection. Furthermore, our analyses do not distinguish which bacterial species harbor specific genes or whether these genes are actively expressed, potentially limiting our understanding of the functional implications. Additionally, the outcomes may be influenced by parameters not included in this study. Finally, the relatively small sample size, limits the statistical power. All results should therefore be confirmed in a larger study material. While fecal samples are commonly used as proxies for the gut microbiome and provide valuable insights into microbial community composition, it is important to acknowledge that they may not fully represent microbial populations throughout the entire gastrointestinal tract [62]. Finally, future analyses should explore functional metabolic pathways in the gut or fecal microbiome; protein family–profiling tools such as ShortBRED [63] could complement the CARD-based results and enable more robust cross-study comparisons, providing deeper insight into how medical treatments shape microbial communities and antimicrobial resistance gene profiles.

## Conclusion

The present study reveals changes in the abundance of predicted ARGs after approximately one year of non-antimicrobial IBD medical therapy, and different treatment regimens exert different pressures on the resistome. Corticosteroid treatment resulted in less resistance in the microbiota compared to in the persons not treated with corticosteroids. The 5-ASA treatment group revealed the opposite trend; however, as all UC patients in our cohort received 5-ASA, the observed ARG changes could be influenced by UC phenotype rather than treatment alone. Although the findings in our study need to be validated in a larger study population, as an exploratory study our findings provide valuable insights into the potential interplay between AMR and the microbiome in the context of IBD subtypes and medication. An increase in ARGs could compromise antibiotic treatment in IBD patients who are at risk of developing infections due to immunosuppression.

## Supplementary Information

Supplementary Material 1: Supplemental Fig. 1: Medicine use, by diagnosis. Each patient is represented by a row, and medicine use (columns) are indicated by colors. All who use, or have used, immunomodulators, have also used Anti-TNF. Supplemental Fig. 2: Spaghetti plots of significant associations between CARD annotation categories and medicine use. Each patient is represented with a grey line showing the abundance of the annotated category (y axis) at the two timepoints (x axis), stratified by diagnosis groups and medicine use. The black line in the plot shows the change in the average abundances. A-K) Spaghetti plots of ARO Terms and medicine use. L-O) Spaghetti plots of ARM gene families and medicine use. P-AE) Spaghetti plots of Drug Class and medicine use. Supplemental Fig. 3: Spaghetti plots of significant associations between genus abundance and medicine use. Each patient is represented with a grey line showing the abundance of the annotated category (y axis) at the two timepoints (x axis), stratified by diagnosis groups and medicine use. The black line in the plot shows the change in the average abundances. Supplemental Fig. 4: A) Boxplot with individual datapoints of alpha diversity (y axis), by timepoint (x axis) and diagnosis. At inclusion, CD patients had significantly lower alpha diversity than controls (*p*=0.037), but not between UC and controls (*p*=0.68). CD patients had a significant increase from inclusion to follow-up (*p*=0.04), but not UC and controls. B) Boxplot with individual datapoints of alpha diversity (y axis), by timepoint (x axis), diagnosis (color), stratified by medicine use. Paired samples from the same patient are connected with a grey line. The p-values refers to a test for association between medicine use and alpha diversity at follow-up, adjusted for diagnosis and alpha diversity at inclusion (linear regression). Supplemental Fig. 5: Bray Curtis plot on taxa. Pairwise Bray-Curtis dissimilarities were computed for each sample based on the relative abundance of genus. and are here shown as multidimensional scaling plots. Supplemental Table 1: Average reads and average mapped reads. Supplemental Table 2: Counts of reads of the number of terms witch CARD REGI annotations of classes ARO Term, Resistance mechanism, and AMR Gene Family. Supplemental Table 3: Drug Class count data. Supplemental Table 4: Genus count data. Supplemental Table 5: Association between medicine use and total AMR abundance. Supplemental Table 6: Association between medicine use and abundance of ARO terms. Supplemental Table 7: Association between medicine use and abundance of AMR gene families. Supplemental Table 8: Association between medicine use and abundance of Resistance mechanism. Supplemental Table 9: Association between medicine use and abundance of ARG’s annotated as conferring resistance to drug classes. Supplemental Table 10: Association between medicine use and genus abundance.

## Data Availability

Identification of individuals stored in public databases by study code is according to Norwegianlegislation not permitted. It is currently in the secure Service for Sensitive Data database (TSD)at the University of Oslo (https://www.sciencedirect.com/science/article/pii/S1877050921001496). This project is part ofthe IBSEN-III study which is still an ongoing active project. Data is currently being collected andanalyzed by researchers, pertaining to different IBD research questions(https://www.med.uio.no/klinmed/english/research/projects/ibsen-inflammatory-bowel-disease/).Data storage is currently approved until 01.04.2035. Ethical approval and patient consent didnot encompass data sharing, but requests for anonymous data for meta-analyses can beaddressed to corresponding author (AEFM) pending permission from the Regional Committeesfor Medical Research Ethics South East Norway (https://rekportalen.no/#omrek/REK_sor-ost).

## Abbreviations

Ahus: Akershus University Hospital
AMR: Antimicrobial Resistance
ANT: Aminoglycoside nucleotidyltransferase
Anti-TNF: Tumor Necrosis Factor inhibitor
ARG: Antimicrobial Resistance Gene
ARO: Antibiotic Resistance Ontology
ASA: 5- Amino Salicylic Acid
CARD: Comprehensive Antibiotic Resistance Database
CD: Crohn’s disease
CRP: C-Reactive Protein
DNA: Deoxyribonucleic acid
ENA: European Nucleotide Archive
FDR: False Discovery Rate
IBD: Inflammatory Bowel Disease
IBSEN-III: Inflammatory Bowel Disease in South Eastern Norway
INN: Innlandet Hospital Trust
MAPQ: Average Mapping Quality
MLS: Macrolides, lincosamides, streptogramins
NIPH: Norwegian Institute of Public Health
NSC: Norwegian Sequencing Centre
OUS: Oslo University Hospital
OXA: Oxacillin hydrolyzing enzymes
QC: Quality Control
RGI: Resistance Gene Identifier
RNA: Ribonucleic acid
SCCAI: Simple Clinical Colitis Activity Index (SCCAI)
SES: Simple endoscopic score
TTA: Turning the Tide of Antimicrobial resistance
UC: Ulcerative Colitis
