# Supplementary material for: Exploring alterations in the gut resistome in medically treated inflammatory bowel disease patients
Source: BMC Microbiol. 2026 Apr 28;26:547. doi: 10.1186/s12866-026-05101-9 (PMC13255511; doi:10.1186/s12866-026-05101-9)
Supplement: Supplementary file 1 — Supplementary Material 1: Supplemental Fig. 1: Medicine use, by diagnosis. Each patient is represented by a row, and medicine use (columns) are indicated by colors. All who use, or have used, immunomodulators, have also used Anti-TNF. Supplemental Fig. 2: Spaghetti plots of significant associations between CARD annotation categories and medicine use. Each patient is represented with a grey line showing the abundance of the annotated category (y axis) at the two timepoints (x axis), stratified by diagnosis groups and medicine use. The black line in the plot shows the change in the average abundances. A-K) Spaghetti plots of ARO Terms and medicine use. L-O) Spaghetti plots of ARM gene families and medicine use. P-AE) Spaghetti plots of Drug Class and medicine use. Supplemental Fig. 3: Spaghetti plots of significant associations between genus abundance and medicine use. Each patient is represented with a grey line showing the abundance of the annotated category (y axis) at the two timepoints (x axis), stratified by diagnosis groups and medicine use. The black line in the plot shows the change in the average abundances. Supplemental Fig. 4: A) Boxplot with individual datapoints of alpha diversity (y axis), by timepoint (x axis) and diagnosis. At inclusion, CD patients had significantly lower alpha diversity than controls (p=0.037), but not between UC and controls (p=0.68). CD patients had a significant increase from inclusion to follow-up (p=0.04), but not UC and controls. B) Boxplot with individual datapoints of alpha diversity (y axis), by timepoint (x axis), diagnosis (color), stratified by medicine use. Paired samples from the same patient are connected with a grey line. The p-values refers to a test for association between medicine use and alpha diversity at follow-up, adjusted for diagnosis and alpha diversity at inclusion (linear regression). Supplemental Fig. 5: Bray Curtis plot on taxa. Pairwise Bray-Curtis dissimilarities were computed for each sample based on [file 12866_2026_5101_MOESM1_ESM.zip › SupplementalLegends_clean.docx]

**Supplemental Figure 1**: Medicine use, by diagnosis. Each patient is represented by a row, and medicine use (columns) are indicated by colors. All who use, or have used, immunomodulators, have also used Anti-TNF.

**Supplemental Figure 2**: Spaghetti plots of significant associations between CARD annotation categories and medicine use. Each patient is represented with a grey line showing the abundance of the annotated category (y axis) at the two timepoints (x axis), stratified by diagnosis groups and medicine use. The black line in the plot shows the change in the average abundances. A-K) Spaghetti plots of ARO Terms and medicine use. L-O) Spaghetti plots of ARM gene families and medicine use. P-AE) Spaghetti plots of Drug Class and medicine use.

**Supplemental Figure 3**: Spaghetti plots of significant associations between genus abundance and medicine use. Each patient is represented with a grey line showing the abundance of the annotated category (y axis) at the two timepoints (x axis), stratified by diagnosis groups and medicine use. The black line in the plot shows the change in the average abundances.

**Supplemental Figure 4**: A) Boxplot with individual datapoints of alpha diversity (y axis), by timepoint (x axis) and diagnosis. At inclusion, CD patients had significantly lower alpha diversity than controls (p=0.037), but not between UC and controls (p=0.68). CD patients had a significant increase from inclusion to follow-up (p=0.04), but not UC and controls. B) Boxplot with individual datapoints of alpha diversity (y axis), by timepoint (x axis), diagnosis (color), stratified by medicine use. Paired samples from the same patient are connected with a grey line. The p-values refers to a test for association between medicine use and alpha diversity at follow-up, adjusted for diagnosis and alpha diversity at inclusion (linear regression).

**Supplemental Figure 5**: Bray Curtis plot on taxa. Pairwise Bray-Curtis dissimilarities were computed for each sample based on the relative abundance of genus. and are here shown as multidimensional scaling plots.

**Supplemental Table 1:** Average reads and average mapped reads.

**Supplemental Table 2: Counts of r**eads of the number of terms witch CARD REGI annotations of classes ARO Term, Resistance mechanism, and AMR Gene Family.

**Supplemental Table 3:** Drug Class count data

**Supplemental Table 4:** Genus count data

**Supplemental Table 5:** Association between medicine use and total AMR abundance.

**Supplemental Table 6:** Association between medicine use and abundance of ARO terms.

**Supplemental Table 7:** Association between medicine use and abundance of AMR gene families.

**Supplemental Table 8:** Association between medicine use and abundance of Resistance mechanism.

**Supplemental Table 9:** Association between medicine use and abundance of ARG’s annotated as conferring resistance to drug classes.

**Supplemental Table 10:** Association between medicine use and genus abundance.
