# Supplementary figures and images for: Exploring alterations in the gut resistome in medically treated inflammatory bowel disease patients
Source: BMC Microbiol. 2026 Apr 28;26:547. doi: 10.1186/s12866-026-05101-9 (PMC13255511; doi:10.1186/s12866-026-05101-9)

Supplemental Figure 2

# ARO Terms


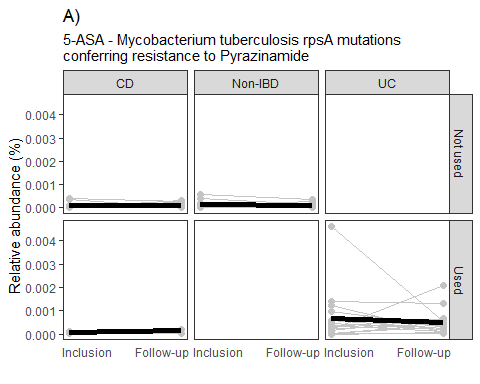

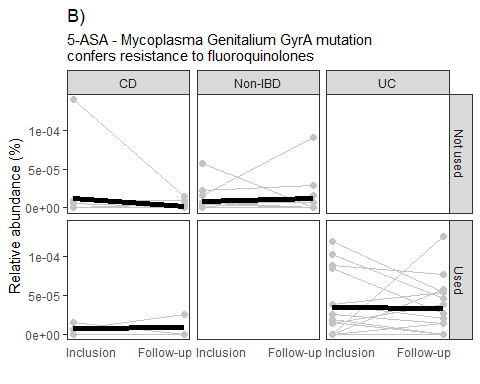

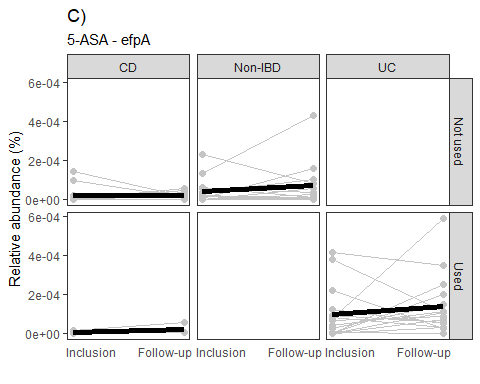

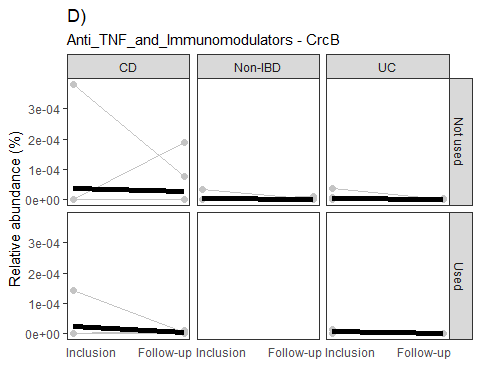

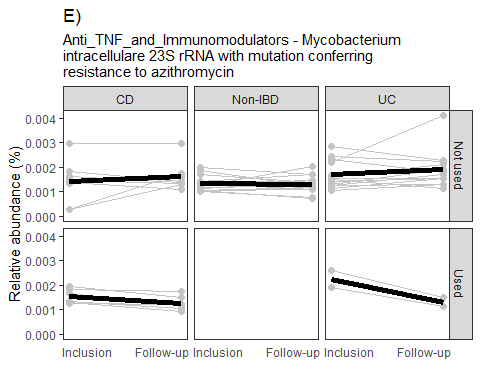

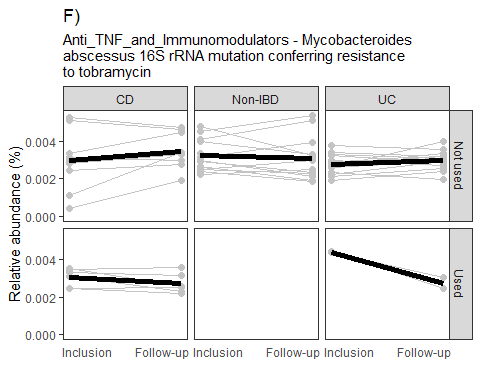

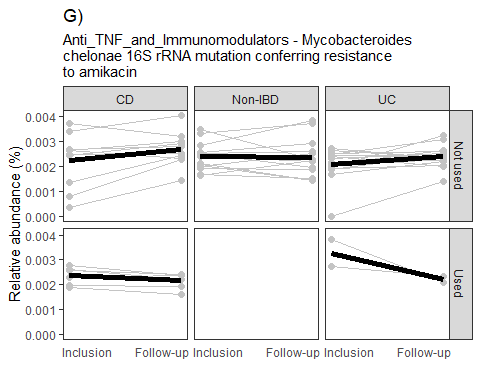

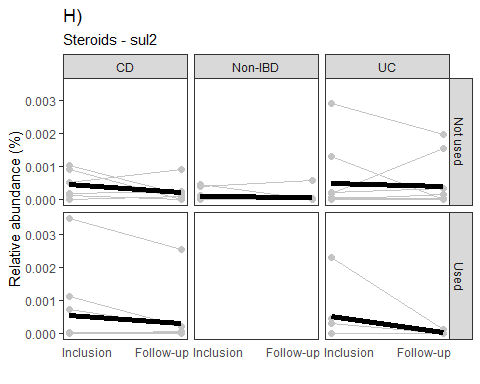


# Gene Families


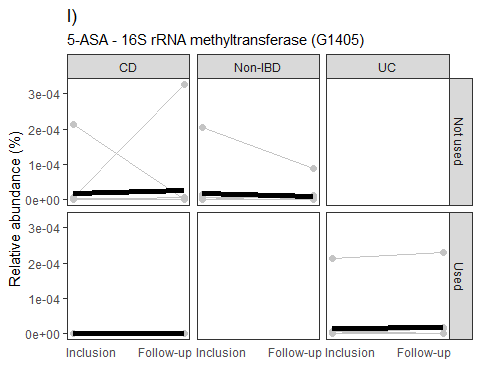

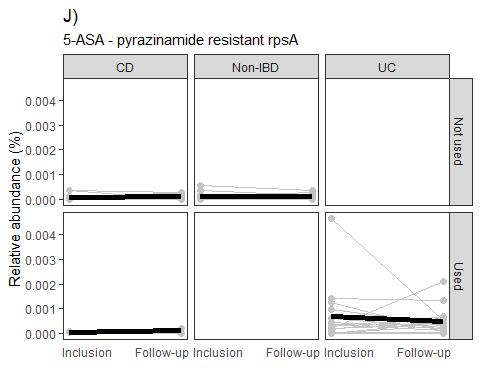

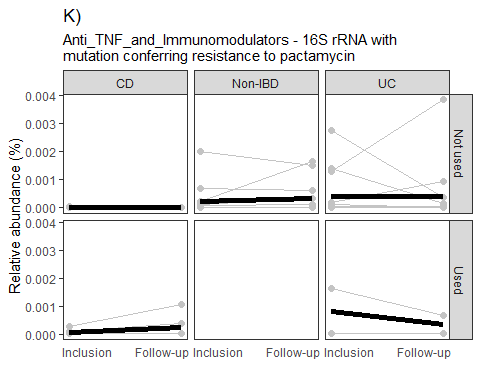

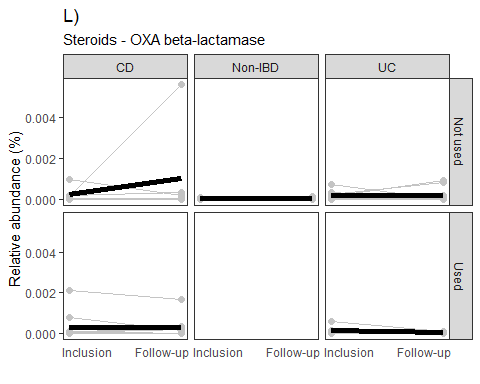


# Drug Class


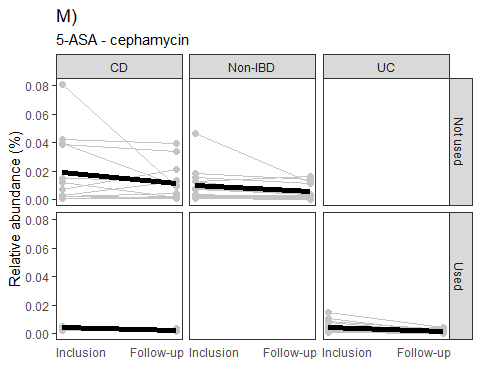

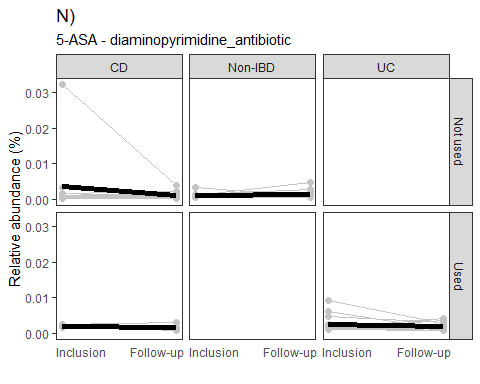

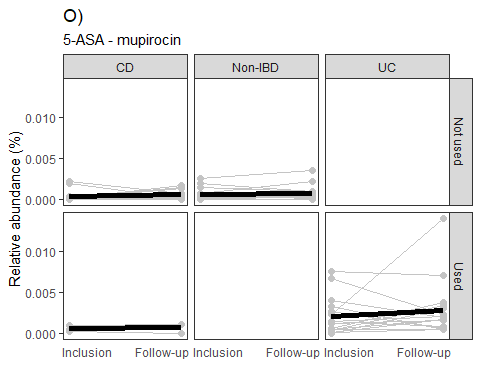

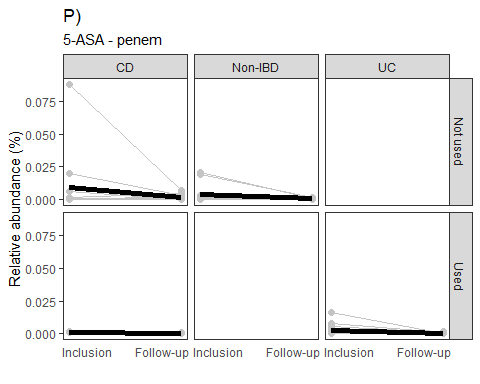

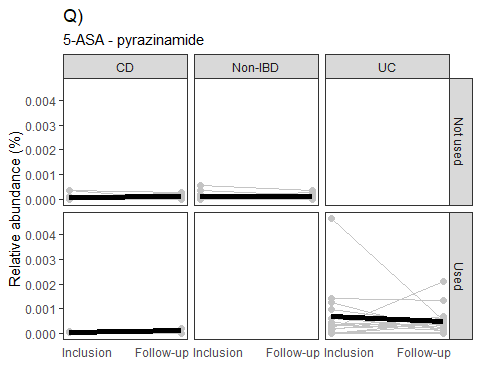

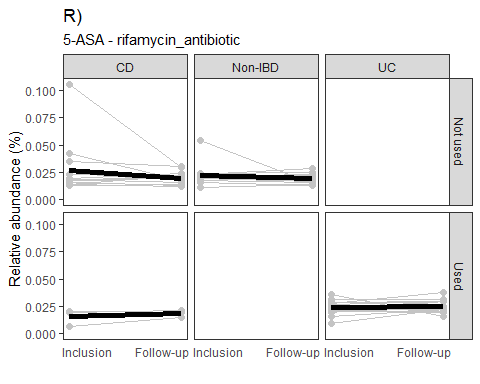

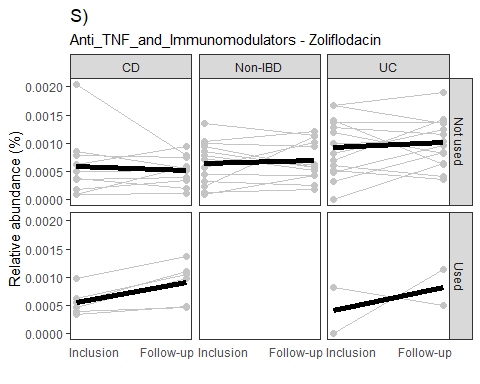

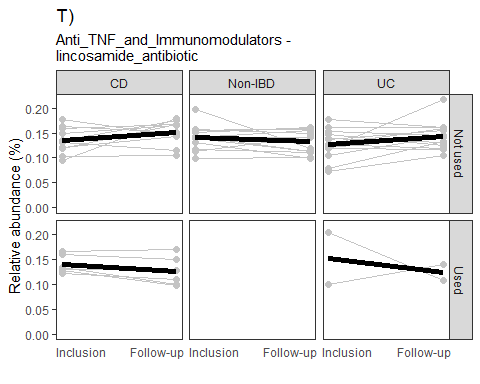

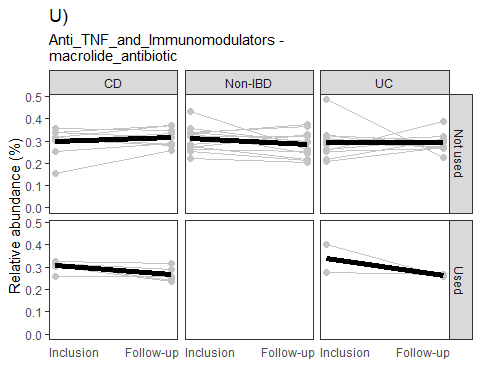

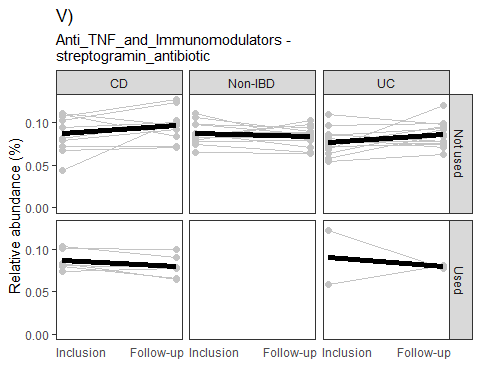

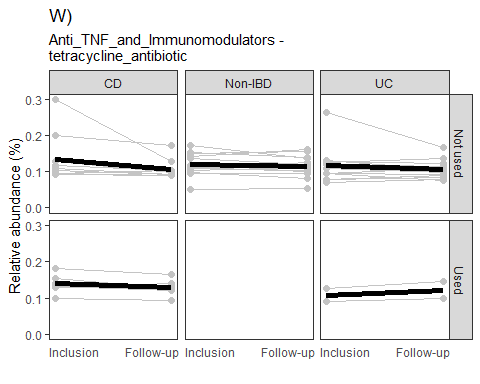

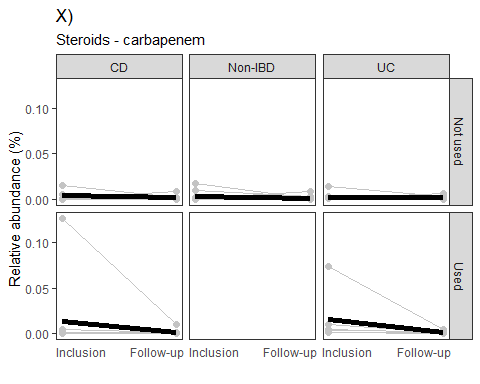

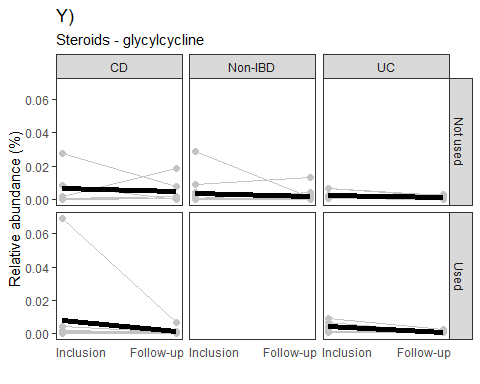

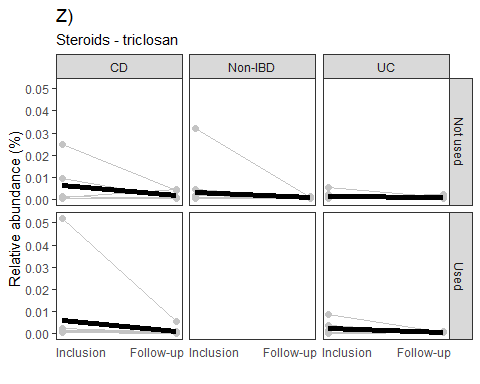

Supplement: Supplementary file 1 — Supplementary Material 1: Supplemental Fig. 1: Medicine use, by diagnosis. Each patient is represented by a row, and medicine use (columns) are indicated by colors. All who use, or have used, immunomodulators, have also used Anti-TNF. Supplemental Fig. 2: Spaghetti plots of significant associations between CARD annotation categories and medicine use. Each patient is represented with a grey line showing the abundance of the annotated category (y axis) at the two timepoints (x axis), stratified by diagnosis groups and medicine use. The black line in the plot shows the change in the average abundances. A-K) Spaghetti plots of ARO Terms and medicine use. L-O) Spaghetti plots of ARM gene families and medicine use. P-AE) Spaghetti plots of Drug Class and medicine use. Supplemental Fig. 3: Spaghetti plots of significant associations between genus abundance and medicine use. Each patient is represented with a grey line showing the abundance of the annotated category (y axis) at the two timepoints (x axis), stratified by diagnosis groups and medicine use. The black line in the plot shows the change in the average abundances. Supplemental Fig. 4: A) Boxplot with individual datapoints of alpha diversity (y axis), by timepoint (x axis) and diagnosis. At inclusion, CD patients had significantly lower alpha diversity than controls (p=0.037), but not between UC and controls (p=0.68). CD patients had a significant increase from inclusion to follow-up (p=0.04), but not UC and controls. B) Boxplot with individual datapoints of alpha diversity (y axis), by timepoint (x axis), diagnosis (color), stratified by medicine use. Paired samples from the same patient are connected with a grey line. The p-values refers to a test for association between medicine use and alpha diversity at follow-up, adjusted for diagnosis and alpha diversity at inclusion (linear regression). Supplemental Fig. 5: Bray Curtis plot on taxa. Pairwise Bray-Curtis dissimilarities were computed for each sample based on [file 12866_2026_5101_MOESM1_ESM.zip › Supplemental Figure 2 AMR spagettiplots.docx]

Supplemental Figure 3


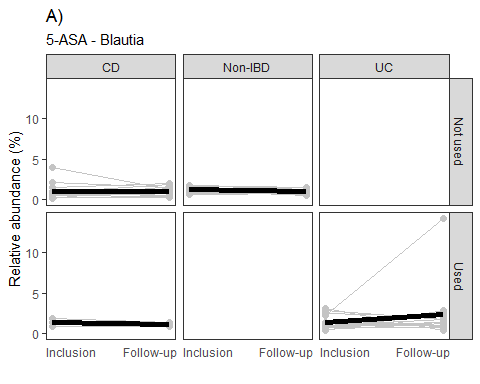

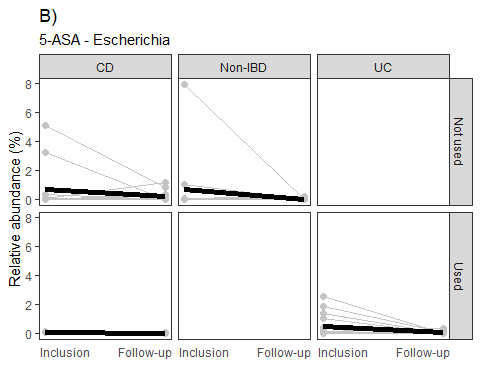

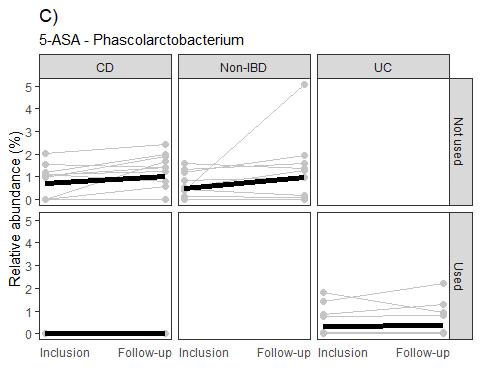

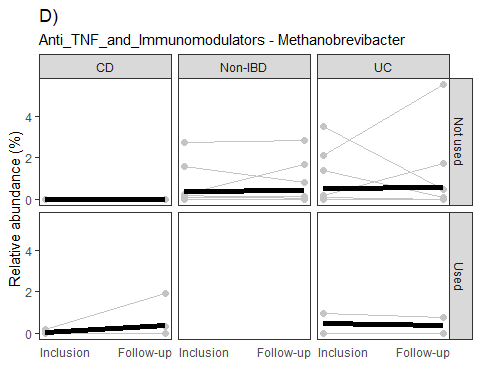

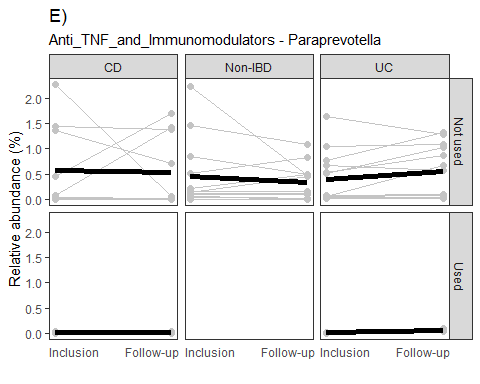

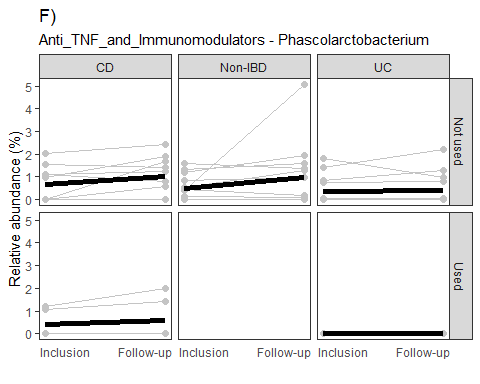

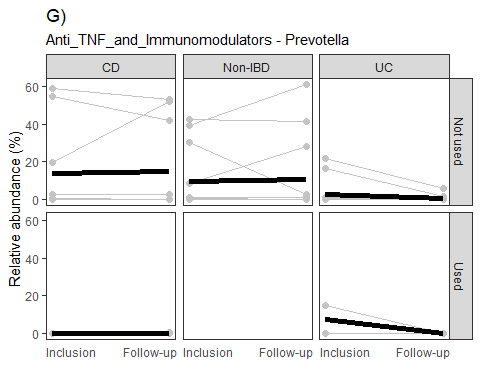

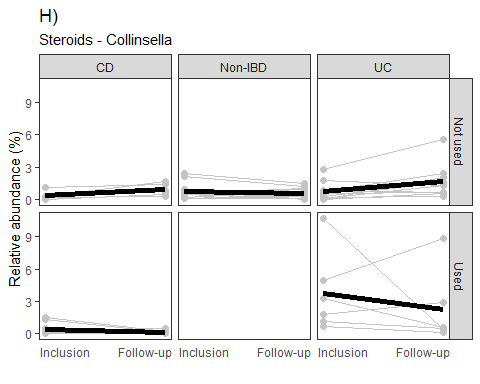

Supplement: Supplementary file 1 — Supplementary Material 1: Supplemental Fig. 1: Medicine use, by diagnosis. Each patient is represented by a row, and medicine use (columns) are indicated by colors. All who use, or have used, immunomodulators, have also used Anti-TNF. Supplemental Fig. 2: Spaghetti plots of significant associations between CARD annotation categories and medicine use. Each patient is represented with a grey line showing the abundance of the annotated category (y axis) at the two timepoints (x axis), stratified by diagnosis groups and medicine use. The black line in the plot shows the change in the average abundances. A-K) Spaghetti plots of ARO Terms and medicine use. L-O) Spaghetti plots of ARM gene families and medicine use. P-AE) Spaghetti plots of Drug Class and medicine use. Supplemental Fig. 3: Spaghetti plots of significant associations between genus abundance and medicine use. Each patient is represented with a grey line showing the abundance of the annotated category (y axis) at the two timepoints (x axis), stratified by diagnosis groups and medicine use. The black line in the plot shows the change in the average abundances. Supplemental Fig. 4: A) Boxplot with individual datapoints of alpha diversity (y axis), by timepoint (x axis) and diagnosis. At inclusion, CD patients had significantly lower alpha diversity than controls (p=0.037), but not between UC and controls (p=0.68). CD patients had a significant increase from inclusion to follow-up (p=0.04), but not UC and controls. B) Boxplot with individual datapoints of alpha diversity (y axis), by timepoint (x axis), diagnosis (color), stratified by medicine use. Paired samples from the same patient are connected with a grey line. The p-values refers to a test for association between medicine use and alpha diversity at follow-up, adjusted for diagnosis and alpha diversity at inclusion (linear regression). Supplemental Fig. 5: Bray Curtis plot on taxa. Pairwise Bray-Curtis dissimilarities were computed for each sample based on [file 12866_2026_5101_MOESM1_ESM.zip › Supplemental Figure 3 genus spagettiplots.docx]

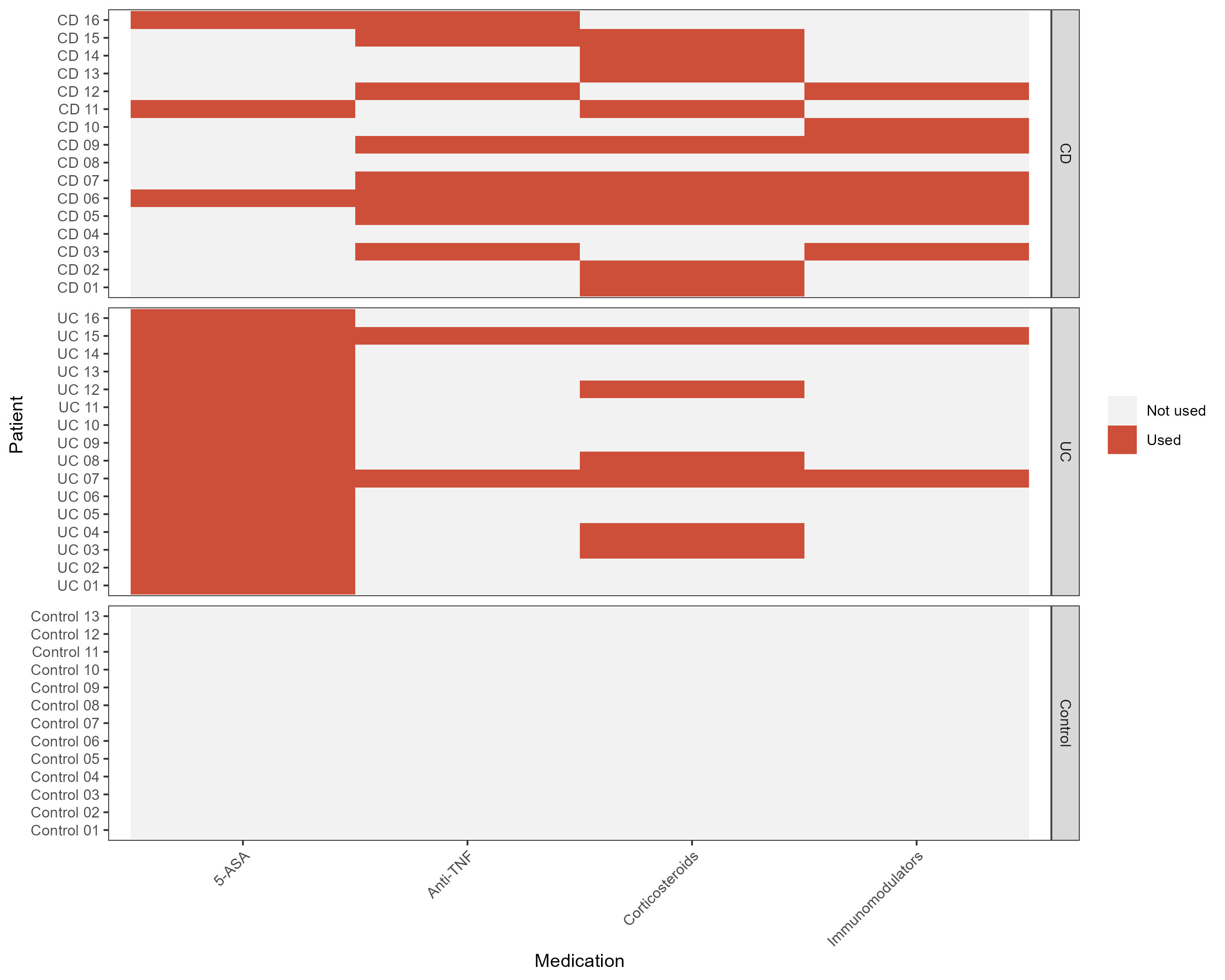

Supplement: Supplementary file 1 — Supplementary Material 1: Supplemental Fig. 1: Medicine use, by diagnosis. Each patient is represented by a row, and medicine use (columns) are indicated by colors. All who use, or have used, immunomodulators, have also used Anti-TNF. Supplemental Fig. 2: Spaghetti plots of significant associations between CARD annotation categories and medicine use. Each patient is represented with a grey line showing the abundance of the annotated category (y axis) at the two timepoints (x axis), stratified by diagnosis groups and medicine use. The black line in the plot shows the change in the average abundances. A-K) Spaghetti plots of ARO Terms and medicine use. L-O) Spaghetti plots of ARM gene families and medicine use. P-AE) Spaghetti plots of Drug Class and medicine use. Supplemental Fig. 3: Spaghetti plots of significant associations between genus abundance and medicine use. Each patient is represented with a grey line showing the abundance of the annotated category (y axis) at the two timepoints (x axis), stratified by diagnosis groups and medicine use. The black line in the plot shows the change in the average abundances. Supplemental Fig. 4: A) Boxplot with individual datapoints of alpha diversity (y axis), by timepoint (x axis) and diagnosis. At inclusion, CD patients had significantly lower alpha diversity than controls (p=0.037), but not between UC and controls (p=0.68). CD patients had a significant increase from inclusion to follow-up (p=0.04), but not UC and controls. B) Boxplot with individual datapoints of alpha diversity (y axis), by timepoint (x axis), diagnosis (color), stratified by medicine use. Paired samples from the same patient are connected with a grey line. The p-values refers to a test for association between medicine use and alpha diversity at follow-up, adjusted for diagnosis and alpha diversity at inclusion (linear regression). Supplemental Fig. 5: Bray Curtis plot on taxa. Pairwise Bray-Curtis dissimilarities were computed for each sample based on [file 12866_2026_5101_MOESM1_ESM.zip › supp_fig_1.png]

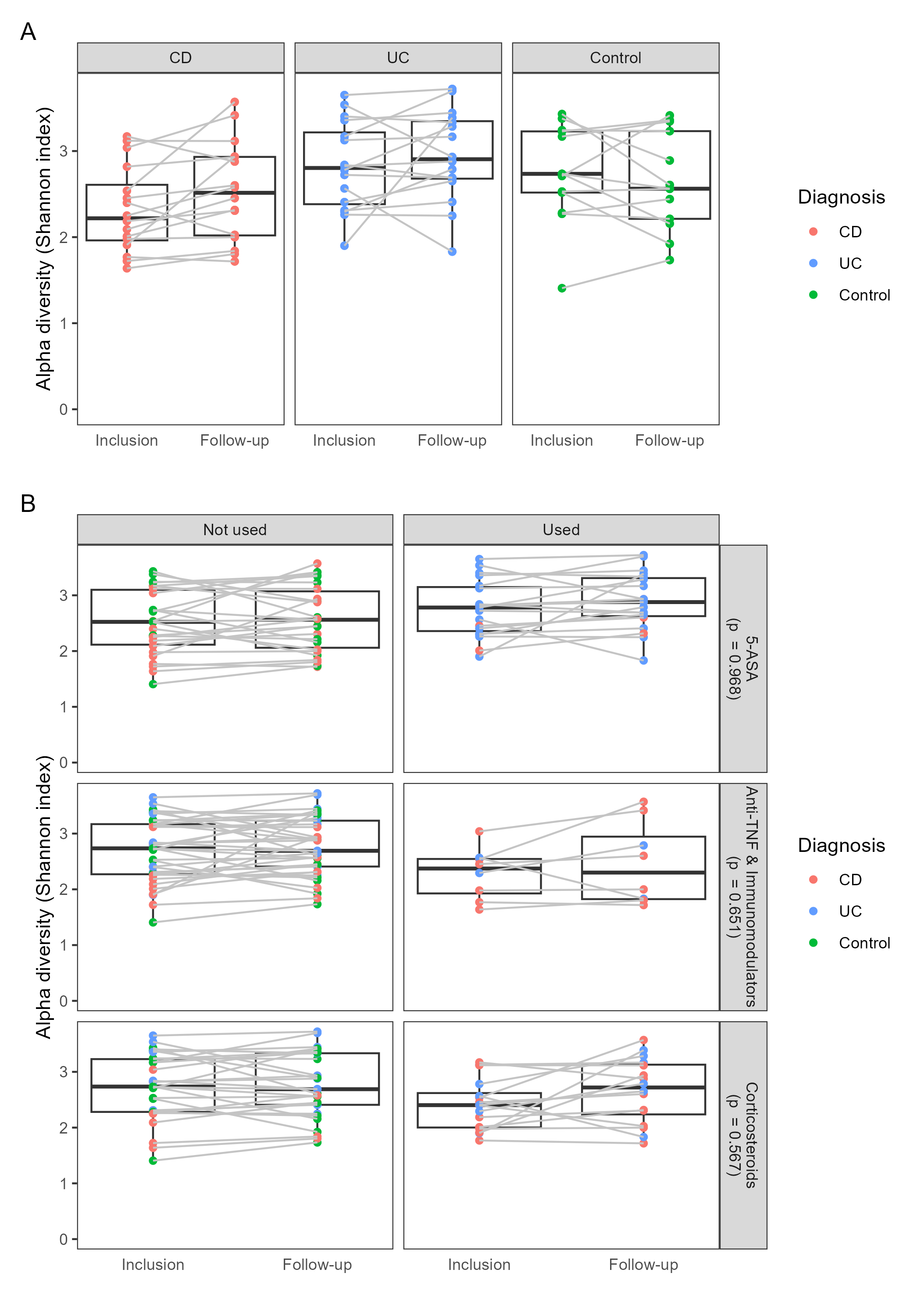

Supplement: Supplementary file 1 — Supplementary Material 1: Supplemental Fig. 1: Medicine use, by diagnosis. Each patient is represented by a row, and medicine use (columns) are indicated by colors. All who use, or have used, immunomodulators, have also used Anti-TNF. Supplemental Fig. 2: Spaghetti plots of significant associations between CARD annotation categories and medicine use. Each patient is represented with a grey line showing the abundance of the annotated category (y axis) at the two timepoints (x axis), stratified by diagnosis groups and medicine use. The black line in the plot shows the change in the average abundances. A-K) Spaghetti plots of ARO Terms and medicine use. L-O) Spaghetti plots of ARM gene families and medicine use. P-AE) Spaghetti plots of Drug Class and medicine use. Supplemental Fig. 3: Spaghetti plots of significant associations between genus abundance and medicine use. Each patient is represented with a grey line showing the abundance of the annotated category (y axis) at the two timepoints (x axis), stratified by diagnosis groups and medicine use. The black line in the plot shows the change in the average abundances. Supplemental Fig. 4: A) Boxplot with individual datapoints of alpha diversity (y axis), by timepoint (x axis) and diagnosis. At inclusion, CD patients had significantly lower alpha diversity than controls (p=0.037), but not between UC and controls (p=0.68). CD patients had a significant increase from inclusion to follow-up (p=0.04), but not UC and controls. B) Boxplot with individual datapoints of alpha diversity (y axis), by timepoint (x axis), diagnosis (color), stratified by medicine use. Paired samples from the same patient are connected with a grey line. The p-values refers to a test for association between medicine use and alpha diversity at follow-up, adjusted for diagnosis and alpha diversity at inclusion (linear regression). Supplemental Fig. 5: Bray Curtis plot on taxa. Pairwise Bray-Curtis dissimilarities were computed for each sample based on [file 12866_2026_5101_MOESM1_ESM.zip › supp_fig_4.png]

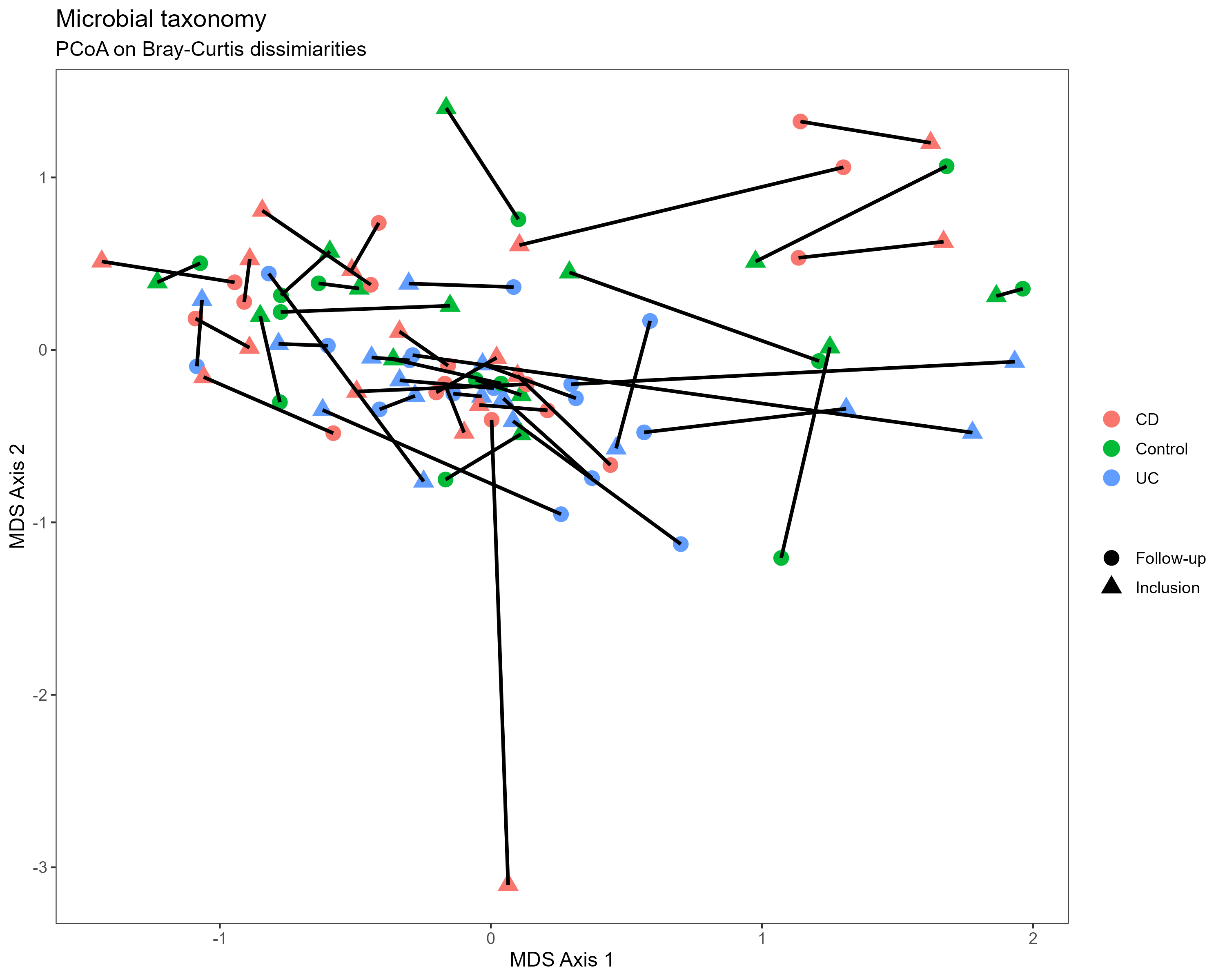

Supplement: Supplementary file 1 — Supplementary Material 1: Supplemental Fig. 1: Medicine use, by diagnosis. Each patient is represented by a row, and medicine use (columns) are indicated by colors. All who use, or have used, immunomodulators, have also used Anti-TNF. Supplemental Fig. 2: Spaghetti plots of significant associations between CARD annotation categories and medicine use. Each patient is represented with a grey line showing the abundance of the annotated category (y axis) at the two timepoints (x axis), stratified by diagnosis groups and medicine use. The black line in the plot shows the change in the average abundances. A-K) Spaghetti plots of ARO Terms and medicine use. L-O) Spaghetti plots of ARM gene families and medicine use. P-AE) Spaghetti plots of Drug Class and medicine use. Supplemental Fig. 3: Spaghetti plots of significant associations between genus abundance and medicine use. Each patient is represented with a grey line showing the abundance of the annotated category (y axis) at the two timepoints (x axis), stratified by diagnosis groups and medicine use. The black line in the plot shows the change in the average abundances. Supplemental Fig. 4: A) Boxplot with individual datapoints of alpha diversity (y axis), by timepoint (x axis) and diagnosis. At inclusion, CD patients had significantly lower alpha diversity than controls (p=0.037), but not between UC and controls (p=0.68). CD patients had a significant increase from inclusion to follow-up (p=0.04), but not UC and controls. B) Boxplot with individual datapoints of alpha diversity (y axis), by timepoint (x axis), diagnosis (color), stratified by medicine use. Paired samples from the same patient are connected with a grey line. The p-values refers to a test for association between medicine use and alpha diversity at follow-up, adjusted for diagnosis and alpha diversity at inclusion (linear regression). Supplemental Fig. 5: Bray Curtis plot on taxa. Pairwise Bray-Curtis dissimilarities were computed for each sample based on [file 12866_2026_5101_MOESM1_ESM.zip › supp_fig_5.png]
